# On the Treatment of Pulmonary Consumption

**Published:** 1855-01

**Authors:** 


' On the Treatment of Pulmonary Consumption.—Many years have not passed since the treatment of pulmonary consumption was almost confined to the palliation of urgent symptoms, and no encouragement was afforded for the expectations we may now occasionally entertain of witnessing permanent amendment under improved methods of management—expectations which may be cherished chiefly in cases subjected to treatment at an early period, and in those instances of advanced disease where cavities are distinctly bounded, and tubercular infiltration does not exist extensively in the lungs.

Even as respects palliative measures there has been of late some accession to our resources. In support of this assertion, let me briefly notice three distressing incidental symptoms—namely night perspirations, diarrhœa, and cough. In the treatment of night perspiration, sulphuric acid was formerly the remedy most commonly employed, and with very partial success. Other acids, such as the gallic and the acetic, are probably more effectual for the purpose. Considerable abatement of the symptoms may often be derived from the use of a night-dress dipped in sea-water or a solution of salt, and dried; but a most important improvement in the treatment of this symptom is the administration of oxide of zinc at bed time, in doses of about four grains, combined with any anodyne extract, such as henbane or hemlock. This property of the oxide was first observed by Dr. Robert Dickson. The mode of action of the remedy is undetermined, but the relief induced by its administration is more decided than any other medicine with which I am conversant.

One of the most trying of the complications of phthisis is diarrhœa. It is not my present intention to attempt a description of the various modifications of this condition, but a few practical remarks on the subject may not be inappropriate. When diarrhœa is associated with intestinal ulceration, or with an appearance of the tongue and other circumstances indicating irritation of the mucous membrane, the diet, notwithstanding the existence of debility, must be bland and unstimulating. Instances dependant on a relaxed state of the intestinal exhalants may require astringents. The peculiarities of the individual case must determine whether we select the preparations of silver, copper, or lead. My experience, however, during the last six years, has confirmed my impression, that of all the remedies which have been recommended for this affection, there is none more generally available than trisnitrate of bismuth. This medicine combined with a little gum and magnesia, and when necessary, with Dover's powder, perseveringly employed, usually affects the object more permanently than other measures, and without inducing any opposite or collateral evils.

The variety of remedies employed for the relief of cough serves to show how intractable this symptom often proves; and I think we may assert that, till within a few years, sooner or later, opium, in some one of its many preparations, has usually become the chief reliance of the practitioner in its treatment. I rarely meet with instances where the inhalation of chloroform, combined with three times the quantity of spirits of wine, fails to relieve; but there are objections to the indiscriminate use of so powerful a measure. Often the inhalation of camphorated spirits is equally effectual; sometimes the vapor of warm water, or infusion of hops. The simple expedient of swallowing a little oil slowly often gives more relief than the use of what are called intentional medicines. In some irritable conditions, tincture of aconite, in doses of four minims, is worth the trial. When expectoration is profuse, creosote, pyro-acetic spirit, infusion of pitch, or balsam of tolu, may be administered; but of all the remedies referable to this class, appropriate for cases accompanied with profuse expectoration, not one has proved in my hands so efficient as petroleum, or Barbadoes tar, the cough being frequently relieved, and the expectoration rapidly moderated, under its use.

On the treatment of hæmoptysis I need not dwell. Single doses of mercury, and a few saline aperients; antimony, even bleeding, may, in special cases, be requisite. Direct astringents, such as gallic acid and acetate of lead, may occasionally be required. In a larger proportion of cases, turpentine is the most satisfactory remedy, but not unfrequently it is inexpedient to attempt the sudden arrest of hæmoptysis, since, when moderate, it is rather useful than dangerous. Such are a few simple hints which I would venture to offer as derived from my own observations of the treatment of individual symptoms. It is not my present object to give an elaborate essay on the treatment of phthisis, but rather to confine my remarks to the statement of opinions either formed or confirmed by my special opportunities for investigation. I feel that a detailed review of the subject would scarcely harmonize with the broad principles which it was the object of preceding lectures to illustrate. The symptoms thus briefly noticed are but incidental evils springing from that error of the organism which I formerly described as exhibiting itself locally in faulty cell-formation, generally in defective blood, and disturbed, imperfect, hurried action. No satisfactory or systematic view can be presented on the subject of treatment without regard to the different degrees of progress of the morbid action.

The disease may be only threatened. Rapid growth, some approach to the phthisical physiognomy, a pearly or somewhat colored margin to the gums, observed in a member of a consumptive family, although not considered an invalid, may be the chief indications of the impending disease. In such instances well-arranged exercise, especially of the arms, as in fencing or archery, the adoption of judicious plans of education, and the appropriate choice of climate or employment, may often avert decided mischief.

But supposing local pulmonary disease to be commencing, as evinced by a disposition to chilliness; a feeling of oppression under the sternum; gnawing sensations about the scapula; effort in making a thorough expiration; prolonged expiratory murmur; hæmoptysis; expectoration, of a character resembling isinglass in appearance; a pulse evincing the characteristics formerly described—congestion should then be obviated. A dose of blue-pill, followed by an aperient, may be administered. Sometimes the application of a few leeches applied over the threatened part, and the administration of liquor potassæ with infusion of cascarilla, may be advantageous.

But softening may occur, the second stage be established, with the clicking, or "humid crepitation," as an auscultatory sign, expectoration exhibiting, perhaps, elastic tissue under the microscope, as well as characteristic cells of tubercular character. The treatment must then be cautious, unstimulating, but strengthening; exposure to cold or heat must be shunned; change to a tropical climate is now dangerous, and even the excitement of sea air may do harm.

When the third stage is established, indicated by gurgling or amphoric respiration or cough, we have to adopt such treatment as shall strengthen the constitution of the patient, so as to become tolerant of the local disorganization, reparation of which can be expected only under very favorable circumstances and assiduous care.

Such general statements as those which I have offered will not, however, satisfy minds anxious to make a distinct and decided step in the treatment of this fatal disease. Do the modern acquisitions furnished by pathology, zco chemistry, and histology, assist us in establishing any principle calculated to determine the appropriate treatment? The chemical condition of the blood of the consumptive patient is, in some respects, analogous to that characterizing chlorosis; but it is not the true olichæmia of that disease and iron will not cure, although it may often be beneficial as an auxiliary.

We have described the deviation from the natural condition in the process of cell-formation as the earliest local manifestation of phthisis. Will our knowledge in this respect assist our decision? Fat degeneration, tubercular degeneration, and cancerous disease, in their cellular relations, may be contrasted. Cancer-cells as compared with phthisical, have, perhaps, more of albumen and less of fat, but the nature of the action is more important than the attendant chemical conditions. In fatty degeneration, we have life failing; in tubercle, running out by undue rapidity; in cancer running riot.

Is there any kind of treatment calculated to moderate this undue action, and at the same time give tone? I have lately tried the influence of digitalis. The effect of this remedy in lowering the pulse is well known; but I have been interested to observe that the rule obeyed by the phthisical pulse is no longer strictly applicable when the sedative influence of digitalis has been produced on the heart. If the anomaly of pulse formerly described depends on the presence of the tubercular element in the blood, the restored susceptibility to change of posture might seem to imply that some effect is produced on the tubercular disease. It is worthy of note that some of the most able physicians of the last century attached great importance to the use of digitalis in pulmonary affections. Dr. Ferriar's remarks on the subject evince an honesty and intelligence which entitle them to our respect. "Digitalis," he observes, "has often quieted the pulse, relieved the cough, and given the patient feelings of recovery." He adds, "the patient's ultimate recovery is not to be confidently expected, even when the pulse is reduced in velocity, and the symptoms are evidently mitigated by the action of the medicine. Many disappointments have taught me not to be elated by one or two instances of success, and I should deceive the public if I presented to them only examples of fortunate practice. To cure (such disorganization as we sometimes observe after death) would require an effort of the power which first created the living body."

It would be unreasonable to disregard the assertion made by so cautious and trustworthy a physician, that, after seven years of careful experiment (from 1792 to 1799) he had come to the conclusion that digitalis, with iron for the scrofulous phthisis, with opium for the florid, enabled him often to hope for a cure under circumstances which would formerly have precluded all hope of recovery. On the whole, we may venture to suggest that the endermic introduction of digitalis, by means of a blistered surface may occasionally prove worth the trial, as an auxiliary measure, at the commencement of some cases of consumption.

The importance of careful and discriminating attention to the peculiarities of each individual constitution must not be overlooked; still there is one medicine so safe and, in a large proportion of cases, so useful, that I shall be excused for giving it a prominent consideration in the remainder of the lecture. Perhaps there is no substance, the value of which has been in a short time established by so large an accumulation of evidence, as cod-liver oil. About ten years since, in my office as one of the Fothergillian adjudicators, some remarks in one of the competing essays, regarding the efficacy of this remedy in scrofula, induced me to try the medicine in consumption. As far as I am aware, the earliest extensive trial of cod-liver oil in London was made by me at the London department of the Hospital for consumption, and the results of that investigation were detailed to this society, the coarse kind, such as is used in tanning leather, being employed. Of the first thirty-seven patients, ten were greatly benefited, and increased materially in plumpness and strength; twelve were slightly improved; twelve remained unchanged; and three were obliged to discontinue the remedy on account of the nausea which it occasioned. Under the use of this oil all the symptoms were ameliorated; the pulse became slower, the cough and expectoration lessened, the night perspirations abated, diarrhœa ceased, and the urine often ceased to exhibit the excess of uric acid. The diminished liability to hectic in patients thus treated furnished remarkable evidence that this variety of fever has more relation to conditions of blood than to local disorganization. It is obviously a question of great practical interest, whether cod-liver oil owes its efficacy to some ingredient not present in other oils. With a view to determine this question I tried various other oils, such as sperm and seal, and the result was a conviction that fish oils generally resembled one another in their remedial properties, although differing in their aptitude for digestive assimilation in the human stomach. It seemed to me an object of interest to determine whether other animal oils not derived from the liver possessed similar powers; and in the year 1849 I made many experiments in the use of neat's-foot oil. Of the first fourteen patients to whom I administered the substance, seven were essentially benefited, (in three the disease was arrested;) in five there was no obvious improvement; only two lost ground. The oil of neat's-foot, therefore, can fairly be compared with that of cod's liver, although, being equally disagreeable to the palate of most patients, it is not likely to come into general use. Dr. Radcliffe Hall, of Torquay, having incidentally heard of this remedy, gave it a trial, and published a favorable account of the effects in the *Monthly Journal* for July, 1852. This physician, not having seen the description of my experiments, his statements furnish an independent confirmation of my conclusions. Neat's-foot oil would appear to be adapted to some cases in which there is too much irritation of the intestinal mucous membrane to bear the administration of the cod-liver oil.

I have also tried an animal oil obtained from a soft, solid fat found between the parchment and the leather skin of animals. The most remarkable properties of this oil, are are its very slight tendency to become rancid, and its freedom from drying properties. It seemed not unreasonable tc expect that it might prove as efficacious as neat's foot oil; but as yet I have not been able to trace any important advantages to its use. I have tried an oil obtained from a species of fish abounding on the Malabar coast, and frequently with encouraging results. The inefficiency of almond and olive oil, excepting when combined with phosphorus, I have already reported. The result of these trials at first induced me to distrust vegetable oils in general: but happening, eighteen months since, to have a patient engaged in a cocoa-nut manufactory, it occurred to me to try the oil of this nut, which, from the nutritious qualities of the kernel, and the peculiar properties of the soap obtained from the oil, seemed worthy of special consideration. In the first instance, I tried the common oil—sometimes the solid, sometimes the fluid part. Subsequently, I have used a purified oil, which is obtained by pressure from crude cocoa-nut oil, as expressed in Ceylon and the Malabar coast from the copperah, or dried cocoa-nut kernel, and refined by being treated with an alkali, which deprives it of its unpleasant smell and taste. It is then repeatedly washed in distilled water, in order to separate any of the saponaceous compound formed by the combination of the alkali with the impurities and rancid matter of the oil. It burns with a feeble blue flame, and is undrying. For the supply of this oil, and for much valuable aid in my investigations, I am indebted to Mr. George Wilson, of Messrs. Price & Co's establishment. Mr. Wilson has given to my suggestions the ready attention of an enlightened mind, and I am happy to bear witness to the value of the sympathy and co-operation which the well-informed laity can often render in our medical endeavors to apply scientific research to purposes of practical usefulness.

Of the first thirty cases of phthisis in which I administered the cocoa-nut oil, nineteen were much improved, five remained without material change, and only six got worse. Of the last twenty-three, fifteen were materially benefited, three remained unchanged and five became worse. Three of these five were in so hopeless a condition at the time of commencing the remedy that my clinical assistant thought it scarcely fair to include them in the report. I was careful, for the most part, to avoid the concurrent use of any other medicine; and it is proper to mention, that a large proportion of the patients had previously taken cod-liver oil, and discontinued it from being disappointed of benefit.

Another oil which I have tried is that of the sunflower seed. It is slightly drying, and burns with a smoky flame, showing a large proportion of carbon. Although the sunflower seed is much used for feeding and fattening poultry, the oil has not, medicinally, afforded me much satisfaction. I have occasionally alternated it with the animal fat oil. Each of these oils h: s occasionally done a little good after the failure of the other, but no results have occurred worthy of record.

I felt curious to ascertain whether chemical science could suggest any explanation of the diversity of remedial properties in different oils, and, with this object, obtained from Mr. Dugald Campbell an ultimate analysis, which is subjoined, of some of the oils which I have employed,

ULTIMATE ANALYSIS OF OILS.

| | Carbon. | Hydrogen. | Nitrogen. | Oxygen. |
|---|---|---|---|---|
| Cod-liver oil, | 80·18 | 14·72 | 0.246 | 5·854 |
| Neat's-foot oil, | 64·32 | 12·50 | 0·064 | 23·106 |
| Cocoa nut oil, | 69·62 | 12·49 | 0·060 | 17·830 |
| Olive oil, | 69·38 | 13·47 | 0·058 | 17·092 |

It will be observed that cod-liver oil differs materially from the other oils in its elementary composition. The remarkable difference apparent between the proportions of these elements, especially of carbon and oxygen, in cod liver oil, as contrasted with the other oils, might lead us to suppose that its superiority might thus be explained; but the inadequacy of such an explanation becomes obvious, when we further pursue the inquiry, and observe a close correspondence in elementary composition between olive oil, which may be regarded, when introduced into the stomach, as medically, almost inert, and cocoa-nut oil, which is fairly comparable in efficacy with the oil obtained from the cod.

I have elsewhere supplied evidence, that the oils which I have chiefly employed evidently modify the condition of the blood. It It is perhaps in this way that they accomplish more in correcting the morbid element on which phthisis depends, at whatever period administered, than any other remedy with which we are at present conversant. Can they be advantageously introduced by friction? For the last six years I have frequently adopted this plan, sometimes as a substitute, at other times as a supplement to the internal administration of the remedy, and often with excellent effects. In the first patient with whom I adopted this plan six years since, who was affected with vomica and exhausted by diarrhœa, the principal treatment consisted in rubbing in an ounce of the oil flavored with lavender night and morning. He was restored to average health, and some years afterwards died of another disease. It is very rare that any measure of real value proves absolutely novel. I had till lately overlooked the fact that Aretæus, Cœlius Aurelianus and Celsus, all speak of inunction with oil in phthisis.

Even neat's-foot oil at the commencement of this century was used by a quack named Samuel Braunston of Shuckburgh, in Warwickshire, for the cure of every disease, and cocoa-nut paste is used in the East for fattening poultry, although, as far as I am aware, its medical efficacy was hitherto unknown. I cannot believe, however, that the oil acts exclusively in the way of supplying nutriment, since on the use of a quarter of a pint a week a patient will often gain one or two pounds in weight. It seems to modify the condition of the granules which enrich the blood, and to dispose them to the calm progression of change by which they are made to contribute to the production of healthy structure. I have seen an ulcer with a surface of deficient granulations brought speedily into a healthy healing condition by the daily application of a drop or two of cod-liver oil. Let a few granules be brought into healthy action, and the mass of granules seem to adapt themselves to the law of improvement. The part of the ulcer not directly in contact with the oil is still modified by its influence, and I have sometimes been induced to suppose that something analogous to the contagiousness of action, (to use an expression of Liebig, as applied to some chemical phenomena,) must be concerned in such results. Let me mention, as an instance of this action, that nitric acid will not readily influence platinum; but if this metal be mixed with silver, the action which is immediately induced by the acid on the latter is soon extended to the platinum.

It is a popular, and probably correct, opinion amongst whale-voyagers, that their expeditions are a cure for consumption. The quantity of oil under such circumstances drunk and introduced by the skin may contribute to a result which indeed may be further promoted by their active exercise and exposure to the air. In some hunting countries great efficacy is attached to drinking the blood from the jugular vein of a young doe. In both these instances collateral circumstances must be included in the consideration, and the same remark will apply to the experience of various observers, adduced by Dr. Simpson, of the comparative immunity from scrofula and phthisis enjoyed by those who work at certain manufactories, where oil, being largely employed, is believed to be introduced into the system of the workpeople through the lungs and the skin; but the statement is well deserving of careful and extensive investigation. Neat's-foot and cocoa oil answer well endermically, and are not offensive. In conducting a course of oil treatment we must not assume that the oil can be indiscriminately used without regard to the condition of the system, and more especially of the mucous membrane. If a state of erethism exists, the preliminary administration of salines and prussic acid will often be desirable. Since the introduction of cod-liver oil, consumption has certainly been divested of many trying incidents; the progress of the disease is often long delayed, and the final sinking stage abridged. Often the fatal termination is ushered in by a sudden attack of congestion. I am inclined to think that this condition might occasionally be obviated, and that when the pulse has been for some time gaining strength, and the patient's weight increasing, the oil should occasionally be suspended, aperients administered, and, should any sensation of thoracic oppression be experienced, cupping-glasses applied. Such measures, when well-timed, rather promote than counteract the appropriate effects of the oil.

Let us not forget that the class of remedies we are considering has no exclusive adaptation to consumptive disease, but probably produces its good effects by correcting a condition which, in some degree, appertains to other disorders—for example, to anæmia in its various forms, whether associated with neuralgia, hypochondria, or hysteria, and, above all, that it is particularly suited for children of scrofulous constitution.

If hereditary tendency to phthisis exists in any family, it is surely of great importance to anticipate the pulmonary era, and introduce oleaginous medicines at an early period. The practice of daily inunction with preparations of neat's-foot or cocoa oleine might, I conceive, prove of peculiar efficacy. Were there time I might be tempted to dwell on this subject as affording a practical illustration of a position with which I began my first lecture, viz: that special observation is chiefly useful by conducting us securely to general laws. In the knowledge of laws dwells practical power and prophetic aptitude. It is by cautious experiment and scientific method we may hope to dissipate the mists of theory, and attain to discoveries sought in vain by the blind and guileless industry of former days.

In conclusion, gentlemen, allow me to say that your courteous attention to the lectures which I have had the honor to deliver will be a subject of grateful recollection in my future career. A motive to continued exertion is supplied by the example and sympathy of so many eminent members of this Society, thus willing to leave the attractions of domestic repose or of profitable engagements, in order to promote the association of scientific research with practical usefulness. It is a privilege to be incorporated with such a fraternity, since men whose minds are habitually exercised for the public good are amongst the greatest boons that Providence has given to the world.—*London Lancet.*

